# Copper Resistance in *Aspergillus nidulans* Relies on the P_I_-Type ATPase CrpA, Regulated by the Transcription Factor AceA

**DOI:** 10.3389/fmicb.2017.00912

**Published:** 2017-05-30

**Authors:** Martzel Antsotegi-Uskola, Ane Markina-Iñarrairaegui, Unai Ugalde

**Affiliations:** Microbial Biochemistry Laboratory, Department of Applied Chemistry, Faculty of Chemistry, University of the Basque CountrySan Sebastian, Spain

**Keywords:** copper resistance, *Aspergillus nidulans*, copper homeostasis, P_I_-type ATPase, transcription factor, metallothionein

## Abstract

Copper homeostasis has been extensively studied in mammals, bacteria, and yeast, but it has not been well-documented in filamentous fungi. In this report, we investigated the basis of copper tolerance in the model fungus *Aspergillus nidulans*. Three genes involved in copper homeostasis have been characterized. First, *crpA* the *A. nidulans* ortholog of *Candida albicans CaCRP1* gene encoding a P_I_-type ATPase was identified. The phenotype of *crpA* deletion led to a severe sensitivity to Cu^+2^ toxicity and a characteristic morphological growth defect in the presence of high copper concentration. CrpA displayed some promiscuity regarding metal species response. The expression pattern of *crpA* showed an initial strong elevation of mRNA and a low continuous gene expression in response to long term toxic copper levels. Coinciding with maximum protein expression level, CrpA was localized close to the cellular surface, however protein distribution across diverse organelles suggests a complex regulated trafficking process. Secondly, *aceA* gene, encoding a transcription factor was identified and deleted, resulting in an even more extreme copper sensitivity than the ΔcrpA mutant. Protein expression assays corroborated that AceA was necessary for metal inducible expression of CrpA, but not CrdA, a putative metallothionein the function of which has yet to be elucidated.

## Introduction

Copper (Cu) is an essential trace element that functions as cofactor of enzymes involved in a wide range of biochemical processes including cellular respiration (cytochrome-c oxidase), free radical detoxification (superoxide dismutase), pigmentation (tyrosinase), and collagen maturation (lysyl oxidase; Lutsenko, 2010; Nevitt et al., 2012). However, excess accumulation of copper promotes the generation of hydroxyl radicals that induce severe cellular damage (Halliwell and Gutteridge, 1990; Valko et al., 2005). Copper toxicity can also be triggered by displacement of other metals present in molecules leading to protein dysfunction (Macomber and Imlay, 2009; Lemire et al., 2013).

Because environmental copper availability may fluctuate in ecological niches (soil, pollutants, etc.), all biological systems have developed homeostatic mechanisms that sense copper levels and respond in order to maintain adequate cellular concentrations within the required threshold levels. To ensure this balance, cells tightly control copper uptake, cellular traffic, storage, and detoxification (reviewed in Nevitt et al., 2012).

In the model organism *Saccharomyces cerevisiae*, elevated environmental copper concentrations (>1 μM) result in a copper-dependent inactivation of the transcription factor (TF) ScMac1p (Graden and Winge, 1997). In consequence, the genes coding proteins implicated in copper uptake, two high affinity Cu^+^ transporters (ScCtr1p and ScCtr3p) and a surface Cu^+2^-metalloreductase (ScFre1p), are down-regulated (Graden and Winge, 1997; Labbé et al., 1997; Nevitt et al., 2012). Copper has the opposite effect on the transcriptional factor ScAce1p (Keller et al., 2005; Ehrensberger and Bird, 2011). Formation of a tetra-copper cluster in the regulatory domain of the TF results in a conformational change that leads to the activation of the inactive Apo-ScAce1p (Furst et al., 1988). As a result, ScAce1p is capable to bind the promoters and induce the expression of detoxification genes, *ScCUP1* and *ScCRS5*, which encode two Cu-metallothioneins (MTs; Thiele, 1988; Culotta et al., 1994). Metallothioneins, cysteine (C)-rich low molecular weight polypeptides characterized by high-affinity for diverse metals (Cu, Zn, Cd, Hg, etc.), are found in all eukaryotes and some prokaryotes (Balamurugan and Schaffner, 2006; Sutherland and Stillman, 2011). These proteins are induced in response to exposure to metals, buffering them and lowering their intracellular concentrations.

Other known intracellular copper level regulators are the *P*-type ATPases, which are heavy-metal translocators conserved through all biological kingdoms. These copper extrusion pumps are the major copper resistance determinant in bacteria (Odermatt et al., 1993; Solioz and Odermatt, 1995; Ladomersky and Petris, 2015), while in eukaryotes, in general, they are involved in copper compartmentalization into the secretory network for the subsequent metallation of newly synthesized Cu-dependent enzymes. In humans, hATP7A (MNK, Menkes disease protein) and hATP7B (WND, Wilson disease protein) copper ATPases carry out both, copper delivering and detoxification functions. At basal Cu conditions hATP7A and hATP7B deliver copper to cuproenzymes in the *trans-*Golgi compartment (TGN; Lutsenko et al., 2007) but in response to Cu level increase, ATPase pumps traffic from the TGN to the cell surface in order to efflux copper out of the cytoplasm, thus increasing resistance of cells to this transition metal (Petris et al., 1996; Suzuki and Gitlin, 1999). In contrast, in *Candida albicans*, each function is achieved by different P_I_-type ATPases; CaCcc2p is involved in cuproenzyme biosynthesis (Weissman et al., 2002) while CaCrp1p is committed to copper export (Riggle and Kumamoto, 2000; Weissman et al., 2000). Indeed, CaCrp1p is mainly responsible for the unusual high copper tolerance of this dimorphic yeast, in contrast to other eukaryotes were copper detoxification relies significantly on MTs. Recently, it has been shown in *A. fumigatus* that histidine is also involved in copper detoxification (Dietl et al., 2016).

The great majority of studies in copper homeostasis have focused on mammalian, bacteria, and yeast cells, but not in filamentous fungi, a major group of microorganisms which includes species of agricultural, food, clinical and environmental interest. *Aspergillus nidulans* is one of the best characterized fungal species and offers the opportunity to study in detail the precise mechanisms involved in Cu balance. The present report describes the identification and characterization of the major copper resistance determinant in *A. nidulans*, the copper transporting P_I_-type ATPase, CrpA. We show that CrpA expression is highly inducible and dynamic in response to prolongued copper exposure and localizes close to the cellular surface as a result of an organized trafficking process. In addition, we demonstrate that metal-dependent induction of CrpA is under the control of AceA, a transcription factor activator of genes involved in copper detoxification. A third gene encoding a metallothionein-like protein has been identified, CrdA, although further studies are needed to elucidate its function. Our results indicate that copper detoxification in *A. nidulans*, as in the dimorphic fungus *C. albicans*, relies mainly in copper excretion.

## Materials and methods

### Bioinformatic

Alignments were performed using the predicted protein sequences released in the National Centre for Biotechnology Information (NCBI) database. Multiple sequence alignments were performed using BLAST and Clustal Omega application in EBI (http://www.ebi.ac.uk/Tools/msa/clustalomega/). Alignments visualization and domain analysis were performed with Jalview program. Transmembrane domains were predicted using Hidden Markov Models (HMM) in the Institute Pasteur Mobyle server (http://mobyle.pasteur.fr/).

### Strains, media, and growth conditions

*A. nidulans* strains used in this study are listed in Table 1. Strain MAD1427 was used as a recipient for transformation and generation of single/double-null and GFP/HA_3_-tagged strains. Appropriately supplemented Käfer's minimal (MMA) and complete (CMA) pH 6.8 buffered medium (Käfer, 1965) containing 1% (w/v) D-glucose and 71 mM sodium nitrate as main carbon and nitrogen source was used to cultivate *A. nidulans*. Liquid culture experiments were conducted with MMA without agar (liquid MMA). General molecular techniques followed (Sambrook et al., 1989).

**Table 1:** ***Aspergillus nidulans* strains used in this study**.

**Strains**	**Genotype**	**References**
MAD1427	*pyrG89, pabaA1; argB2; ΔnkuA::argB; veA1, riboB2*	TN02A25 Oakley B.
MAD2731	*pabaA1; argB2; ΔnkuA::argB; veA1, riboB2*	Markina-Iñarrairaegui et al., 2011
MAD2733	*pabaA1; argB2; ΔnkuA::argB; veA1*	Markina-Iñarrairaegui et al., 2011
BD888	*pyrG89, pabaA1; argB2; ΔnkuA::argB; ΔcrpA::pyrG^*Af*^; veA1, riboB2*	This study
BD892	*pyrG89, pabaA1; argB2; ΔnkuA::argB; crpA::gfp::riboB^*Af*^; veA1, riboB2*	This study
BD894	*pyrG89, pabaA1; argB2; ΔnkuA::argB; crpA::ha_3_::pyrG^*Af*^; veA1, riboB2*	This study
BD896	*pyrG89, pabaA1; argB2; crdA::ha_3_::pyrG^*Af*^, ΔnkuA::argB; ΔcrpA::riboB^*Af*^; veA1, riboB2*	This study
BD898	*pyrG89, pabaA1; argB2; ΔcrdA::pyrG^*Af*^, ΔnkuA::argB; veA1, riboB2*	This study
BD900	*pyrG89, pabaA1; argB2; crdA::ha_3_::pyrG^*Af*^, ΔnkuA::argB; veA1, riboB2*	This study
BD961	*pyrG89, pabaA1; argB2; ΔnkuA::argB; crpA::ha_3_::pyrG^*Af*^; ΔaceA::riboB^*Af*^; veA1, riboB2*	This study
BD963	*pyrG89, pabaA1; argB2;ΔcrdA::pyrG^*Af*^, ΔnkuA::argB; ΔcrpA::riboB^*Af*^; veA1, riboB2*	This study
BD965	*pyrG89, pabaA1; argB2; ΔnkuA::argB; ΔaceA::pyrG^*Af*^; veA1, riboB2*	This study
BD1062	*pyrG89, pabaA1; argB2; crdA::ha_3_::pyrG^*Af*^, ΔnkuA::argB; ΔaceA::riboB^*Af*^; veA1, riboB2*	This study
BD1073	*pyrG89, pabaA1; argB2; ΔnkuA::argB; crpA^*An*^ (ΔcrpA::pyrG^*Af*^); veA1, riboB2*	This study

Colony growth tests were carried out by inoculating conidiospores on solid MMA and incubating for 48 h at 37°C. Phenotypes caused by the deletion of genes were studied under an array of metal stresses induced by addition of CuSO_4_ (50, 100, 150, 200, 400, 600 and 1000 μM), AgNO_3_ (2.5 and 5 μM) and Cd(NO_3_)_2_ (100, 150, and 250 μM). Radial extension and colony morphology of mutant strains was always compared with that of the wild-type MAD1427 strain. Close-up views of colonies grown for 36, 48, and 72 h at 37°C were observed and photographed with a binocular microscope (Nikon SMZ800).

To quantify the effect of CuSO_4_, AgNO_3_, and Cd(NO_3_)_2_, on biomass production liquid culture experiments were carried out. 1.10^6^ spores were inoculated in liquid MMA (30 ml medium in 100 ml flask) with or without metal agents and incubated for 24 h at 37°C in a rotary incubator at 200 rpm. The cultures were collected, dried for 24 h at 100°C, and their dry weight measured. Three technical replicates were performed. Two-tailed Student's *t*-test for unpaired samples was used for the statistical analysis to compare the cellular growth between two strains in different conditions.

RNA and protein extracts were isolated from mycelium of strains cultivated in a fermenter for 16 h at 37°C. Cells were harvested before (0 min) and after adding the metal agent to fresh MMA and incubating for the period indicated in the figures. Mycelia was collected by filtration through Miracloth (Calbiochem), squeezed to dry and frozen in liquid nitrogen. Samples for protein extraction were lyophilized for 16 h.

### Generation of null and tagged strains

Genomic cassettes for the generation of strains carrying null alleles and GFP or HA_3_ C-terminally tagged fusion proteins were constructed following fusion-PCR technique described in Markina-Iñarrairaegui et al. (2011). Null mutants were generated by using deletion cassettes containing the *Aspergillus fumigatus pyrG* or *riboB* as prototrophic selection marker flanked by 1,500 bp of 5′ UTR and 3′ UTR regions of the target gene. Firstly, each fragment was amplified using specific oligonucleotide pairs; gsp1–gsp2 (5′ UTR), gsp3–gsp4 (3′ UTR), and gsp2^*^–gsp3^*^ (selectable marker). The gsp2^*^, gsp3^*^, and gsp6^*^ (see below) hold, in addition of the selectable marker sequence, a 24 bp tail homologous to the gsp2, gsp3, and gsp6 gene specific primers. The fragments containing the selectable marker were amplified using a plasmid as a template. Afterwards, the three fragments were fused using gsp1-gsp4 primers. For the C-terminal tagging *ha*_3_::*pyrG*^*Af*^ and *gfp::riboB*^*Af*^ fragments, the 3′ end (~1,500 bp) of the gene and the 3′ UTR regions were amplified using oligonucleotides gsp6^*^–gsp3^*^, gsp5–gsp6, and gsp3–gsp4, respectively. Finally, the tagging cassettes were constructed using gsp5 and gsp4 oligonucleotides to fuse the fragments. Oligonucleotides used in this study are summarized in Table 2.

**Table 2:** **Oligonucleotides used in this study**.

**Oligo**	**Sequence (5′–3′)**
CrpA-gsp1	CGTACATGGGTCTGGTCTTCCCC
CrpA-gsp2	GACGAGTGGCGGCTAGTGTTCC
CrpA-gsp3	TTATTTTTTTCTAGTTCCATGCATGC
CrpA-gsp4	CCCTGAGCAGTCTCGATGAG
CrpA-gsp5	CTTCAGCGGGTCGCAGATACG
CrpA-gsp6	CTCCTGTTGACGCGTAGTCCGG
CrpA-gsp2^*^	GGAACACTAGCCGCCACTCGTCACCGGTCGCCTCAAACAATGCTCTTCA
CrpA-gsp3^*^	CATCGCATGCATGGAACTAGAAAAAAATAACTGTCTGAGAGGAGGCACTGATGC
CrpA-gsp6^*^	CCGGACTACGCGTCAACAGGAGGGAGCTGGTGCAGGCGCTGGAGCC
CrdA-gsp1	GGCTTCGAGAACTACCAGAACC
CrdA-gsp2	ATTGAATGTTGTTTGAATGGTAG
CrdA-gsp3	TGCGTTTGAATTCATGTTAATGAAGC
CrdA-gsp4	CCAATCCGAGGTCGAGTACG
CrdA-gsp5	ATGGTTCACCCCACCTCAACCTGCT
CrdA-gsp6	AGCCTTGGCCGTCGTAAAATCTGTCTC
CrdA-gsp2^*^	CTACCATTCAAACAACATTCAATACCGGTCGCCTCAAACAATGCTCT
CrdA-gsp3^*^	GCTTCATTAACATGAATTCAAACGCACTGTCTGAGAGGAGGCACTGATG
CrdA-gsp6^*^	GAGACAGATTTTACGACGGCCAAGGCTGGAGCTGGTGCAGGCGCTGGAGC
AceA-gsp1	CCGATGATTCCTTCCACTGCCCAGACATAC
AceA-gsp2	CGCCGCGGTTACTGGGATTGGCACATG
AceA-gsp3	GGACAGCAAGGGCCTTAGAATCTT
AceA-gsp4	ATACAAATAGAGAGGCGAAGGAATGGCG
AceA-gsp2^*^	CATGTGCCAATCCCAGTAACCGCGGCGACCGGTCGCCTCAAACAATGCTCT
AceA-gsp3^*^	AAGATTCTAAGGCCCTTGCTGTCCCTGTCTGAGAGGAGGCACTGAT

The *A. nidulans* transformation procedure followed in this study is an adaptation of the transformable protoplasts production protocol described in Szewczyk et al. (2006) and the protoplast transformation protocol described by Tilburn et al. (1983). The purified genomic casettes obtained above were used to transform *A. nidulans*. Between 7 and 15 transformants were obtained in each regeneration plate (4 plates/transformation). Among the *pyrG*^+^ and *riboB*^+^ transformants, Southern blot technique was used in order to identify homokaryotic recombinant strains carrying a single-copy integration event. Of the initially screened 4 transformants, 2 verified clones were characterized in this study. The double-null mutant and strains combining a null allele and HA_3_-tagged protein were generated by step-by-step transformation with each fusion genomic cassette.

Revertant strain construction was achieved by transforming the null *crpA* strain (BD888) with a DNA cassette containing the *crpA* gene flanked by 1,500 bp up and downstream the corresponding ORF. gsp1 and gsp4 oligonucleotides were used to amplify the cassette using a WT strain genomic DNA as a template. Transformants containing the replacement of the null locus were selected by the ability to resist the 2 mg/ml 5-fluoro-orotic acid (5-FOA; Apollo Scientific, Stockport, United Kingdom) that was added in the medium.

### RNA isolation and northern blot

Total RNA was isolated using TRIzol reagent following the Invitrogen protocol (Invitrogen, Carlsbad, CA) as described in Garzia et al. (2013) and RNA concentration was calculated using a Nanodrop 2000 c system (Thermo Fisher Scientific, Waltham, MA). Ten micrograms of total RNA were loaded in 1.2% agarose gels, transferred to positively charged nylon filters, and analyzed by Northern blot which was carried out with a digoxigenin Northern starter kit (Roche) essentially following manufacturer's instructions and using the hybridization solution (1% BSA, 1 mM EDTA, 0.5 M NaPO_4_, pH = 7.2, and 7% SDS; Church and Gilbert, 1984). Transcript of *crpA* was detected using a genomic probe amplified by PCR using oligonucleotides gsp5 and gsp6 listed in Table 2. Labeling of the probe was performed using DIG High Prime DNA Labeling Starter Kit (Roche) and covered 43% of the ORF. Equal loading of total RNA, which was used as internal control for normalization, was evaluated by ethidium bromide staining of rRNA. Digoxigenin bound probe was detected using CSDP (Roche) in a Chemidoc + XRS (Bio-Rad) system. Intensity of chemiluminescence signal of bands was measured with Image Lab 3.0 software (Bio-Rad).

### Protein isolation and western blot

Protein extraction from lyophilized samples was performed by two different methods. The alkaline-lysis extraction (AL) procedure was used for CrpA extraction, using Lysis buffer (0.2 M NaOH, 0.2% β-mercaptoethanol), as described in Hervás-Aguilar and Peñalva (2010). Protein concentration of extract was estimated loading 5 μl of each sample on polyacrylamide gels. CrdA was extracted as described in Drubin et al. (1988) using Drubin buffer (5 mM HEPES pH 7.5, 1 mM EDTA, 20 mM KCl, 0.1% NP-40, 0.5 mM DTT) supplemented with EDTA-free protease-inhibitor cocktail tablets (1 tablet/50 ml of buffer; Roche). Protein quantification was carried out by Bradford assay (Bradford, 1976) using Bradford dye reagent (Alfa Aesar) and following manufacturer's instructions 12.5 μg of total protein samples were loaded on gels.

Tagged protein expression was analyzed by Western blotting. Proteins were resolved in 8% (CrpA-HA_3_) or 12% (CrdA-HA_3_) SDS-polyacrylamide gels and electrotransfered to Immun-Blot® PVDF membranes by TransBlot® Turbo™ Transfer System (Bio-Rad). HA_3_-tagged proteins were detected using anti-HA mouse antibody [1/10,000 (v/v) dilution for tagged CrpA and 1/1,000 for tagged CrdA detection; Santa Cruz Biotechnology] and CrpA-GFP with mouse anti-GFP (1/5,000; Roche). Hexokinase, used as loading control, was detected with anti-hexokinase rabbit antibody (1/30,000; Chemicon Intemat Inc.). Peroxidase-conjugated goat anti-mouse IgG immunoglobulin (1/4,000 for CrpA and 1/2,500 for CrdA detection; Jackson Immunoresearch Lab) or donkey anti-rabbit (1/10,000; Sigma) cocktails were used as secondary antibodies. Peroxidase activity was detected using Clarity™ Western ECL Substrate (Bio-Rad). Chemiluminescence was observed using a Chemidoc + XRS system (Bio-Rad) and signal intensity was measured with Image Lab 3.0 software (Bio-Rad).

### Fluorescence microscopy

*A. nidulans* conidiospores were cultured in uncoated glass-bottom dishes (Ibidi GmbH, Germany; 2.5 ml of medium per well) for 16 h at 25°C in adequately supplemented pH 6.8 Käfer's minimal medium containing 0.1% D-glucose, 71 mM sodium nitrate and 25 mM sodium phosphate monobasic, similar to watch minimal medium (WMM; Peñalva, 2005). After this period, the medium was replaced with fresh medium supplemented with 100 μM CuSO_4_ to induce CrpA-GFP expression.

Fluorescence images were acquired using a Zeiss Axio Observer Z1 inverted microscope equipped with a 63x Plan Apochromat 1.4 oil immersion Lens, Axiocam MRm Rev.3 camera, an Zeiss HXP 120C external light source for epifluorescence excitation and fitted with filter set 38HE for green fluorescence (Ex BP 470/40; FT 495; Em BP 525/50) and filter set 43HE for red fluorescence (Ex BP 545/25; FT 570; Em BP 605/70). Same exposure time and microscope settings were applied for all image acquirement. Numerous cells were observed for each time before taking representative images. Fluorescence levels were measured using ImageJ software (http://imagej.nih.gov/ij; U.S National Institutes of Health, Bethesda, Maryland, USA).

## Results

### Identification of *Aspergillus nidulans* genes involved in copper homeostasis

In a RNA-seq experiment conducted in our lab (data not published) the expression of the AN3117 gene was significantly increased as a consequence of copper load. Blastp sequence analysis identified the 1,211 amino acid long protein encoded by AN3117 ORF (3,636 bp) as the putative ortholog of *C. albicans* copper-transporting P_I_-type ATPase, CaCrp1p (Riggle and Kumamoto, 2000; Weissman et al., 2000). Based on sequence conservation (35% identity, 55% similarity, and 95% query cover) AN3117 was termed *crpA* (*copper resistance P-type ATPase*). The *A. nidulans* genome contains a second P_I_-type ATPase coding gene, *ygA*, with high homology to CaCcc2p (37% identity; Weissman et al., 2002). YgA is predicted to transport Cu^+^ to the secretory pathway and its absence results in conidial pigmentation impairment (Clutterbuck, 1990).

**Figure 1:**
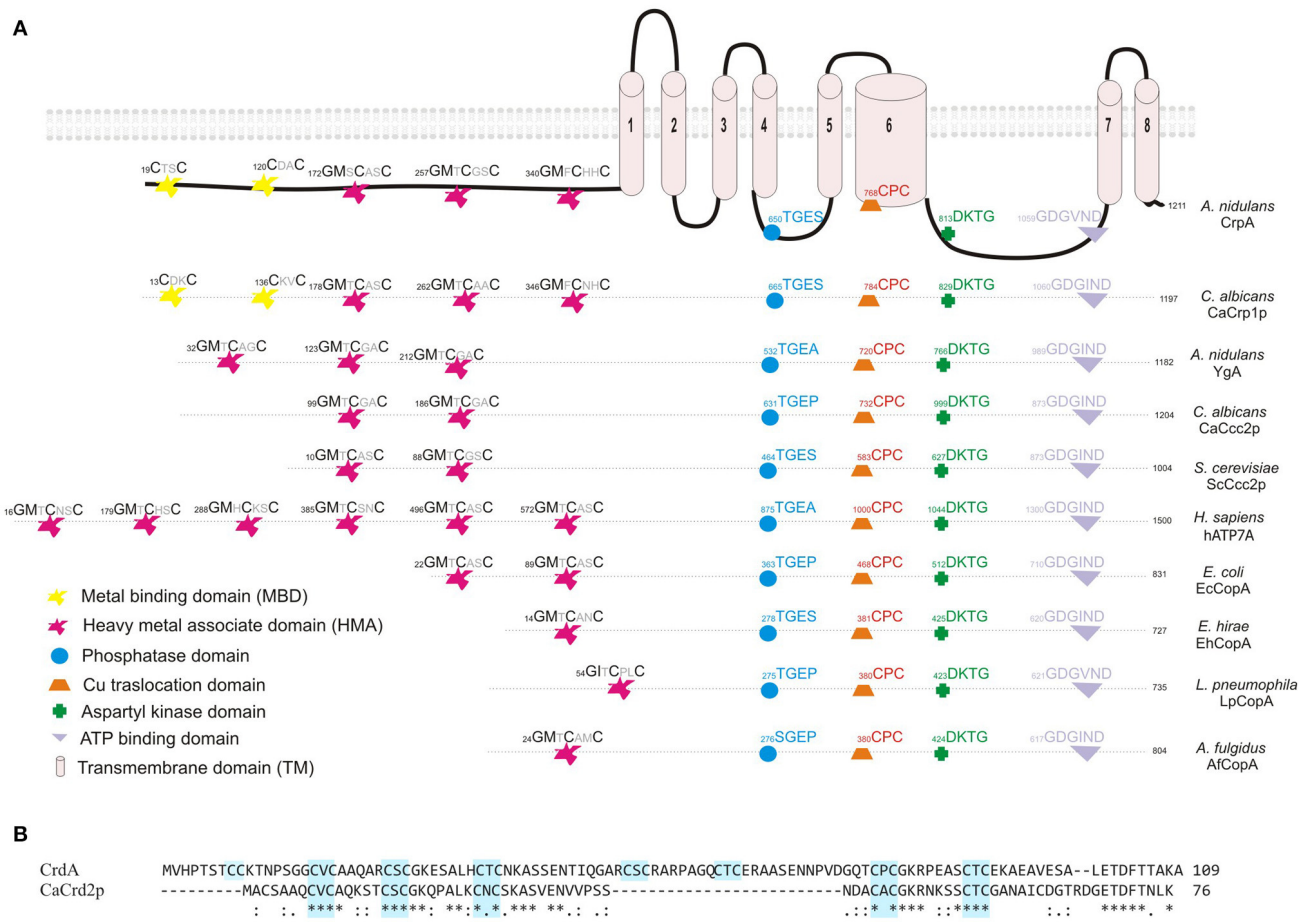
**Sequence analysis of CrpA and CrdA. (A)** Proposed two dimensional model of CrpA describing the predicted membrane topology and comparison of the conserved functional domains of P_I_-type ATPases and their position among different species. GenBank accession numbers are given in parentheses: *Aspergillus nidulans* CrpA (CBF83376.1) and YgA (CBF75750.1); *Candida albicans* CaCrp1p (AAF78958.1) and CaCcc2p (XP_720761.1); *Saccharomyces cerevisieae* ScCcc2p (AAC37425.1); *Homo sapiens* Menkes disease protein hATP7A (AAA35580.1); *Escherichia coli* EcCopA (CDL39203.1); *Enterococcus hirae* EhCopA (AAA61835.1); *Legionella pneumophila* copper-translocating *P*-type ATPase (WP_010947353.1); *Archaeoglobus fulgidus* copper-exporting *P*-type ATPase AfCopA (KUJ93751.1). **(B)** Alignment of predicted full-length of CrdA and CaCrd2p sequences compared using Clustal method. Asterisks describe identical, *double* and *single dots*, conservative and semi-conservative residues, respectively. CxC repeats are boxed in light blue. Protein accession numbers are reported as follows: *Aspergillus nidulans* CrdA (CBF79264.1) and *Candida albicans* CaCrd2p (AAF78959.1).

As shown in Figure 1A, bioinformatic analyses predicted in CrpA the presence of the distinctive domains described for well-studied copper-transporting ATPases of yeast, human, bacteria and archaea (Solioz and Odermatt, 1995; Mandal et al., 2002; Barry et al., 2010; Rosenzweig and Argüello, 2012; Migocka, 2015; Smith et al., 2016; Figure 1A); 8 transmembrane domains (TM), a conserved CPC copper translocation motif placed in the 6th TM segment and cysteine rich metal binding motifs (MBD) in the cytoplasmic N-terminal. 5 N-MBD are predicted as tandem repeats, 2 CxxC motifs located closer to the amino terminus followed by 3 GMxCxxC classical heavy metal associated domains (HMA). This structure of Cu-binding domains in the NH_2_-terminal extension is similar to *C. albicans* CaCrp1p (Riggle and Kumamoto, 2000; Weissman et al., 2000) but different from other homologs. In common with non-heavy metal P-type ATPases, CrpA shares characteristic features as an aspartyl kinase domain (DKTG) in a large cytoplasmic loop containing an aspartate residue that is transiently phosphorylated during the catalytic cycle, a phosphatase domain (TGES) and a consensus domain for ATP binding and energy transduction (GDGVNDSP; Palmgren and Nissen, 2011; Inesi et al., 2014).

Earlier studies had shown that metallothioneins (MTs) play a significant role in copper tolerance. For this reason, *in silico* searches were carried out using *S. cerevisiae* (ScCup1p and ScCrs5p) and *C. albicans* (CaCup1p and CaCrd2p) sequences. Protein database searches revealed the existence of a single putative MT-like protein in *A. nidulans*, product of AN7011 and possible ortholog of Ca*CRD2*, named *crdA*. The 109 residues of CrdA are 44% similar and 36% identical to (84% query cover) CaCrd2p. CrdA is longer and has additional cysteine residues, which is not surprising due to the diversity in primary structure of this heterogeneous protein family (reviewed by Blindauer and Leszczyszyn, 2010). As shown in Figure 1B the 16 cysteine residues are scattered throughout the entire sequence, arranged in cysteine clusters which are essential for copper binding. CrdA contains seven CxC clusters of which four shared the location of all CxC clusters identified in CaCrd2p. Besides, CrdA shares signature features of MTs; few aromatic amino acids (1 aa), lack of histidine and high Ser and Lys residue content (9 and 6%, respectively; Riggle and Kumamoto, 2000). Additional searches did not reveal any further known conserved functional domains.

### Functional analysis of CrpA and CrdA

**Figure 2:**
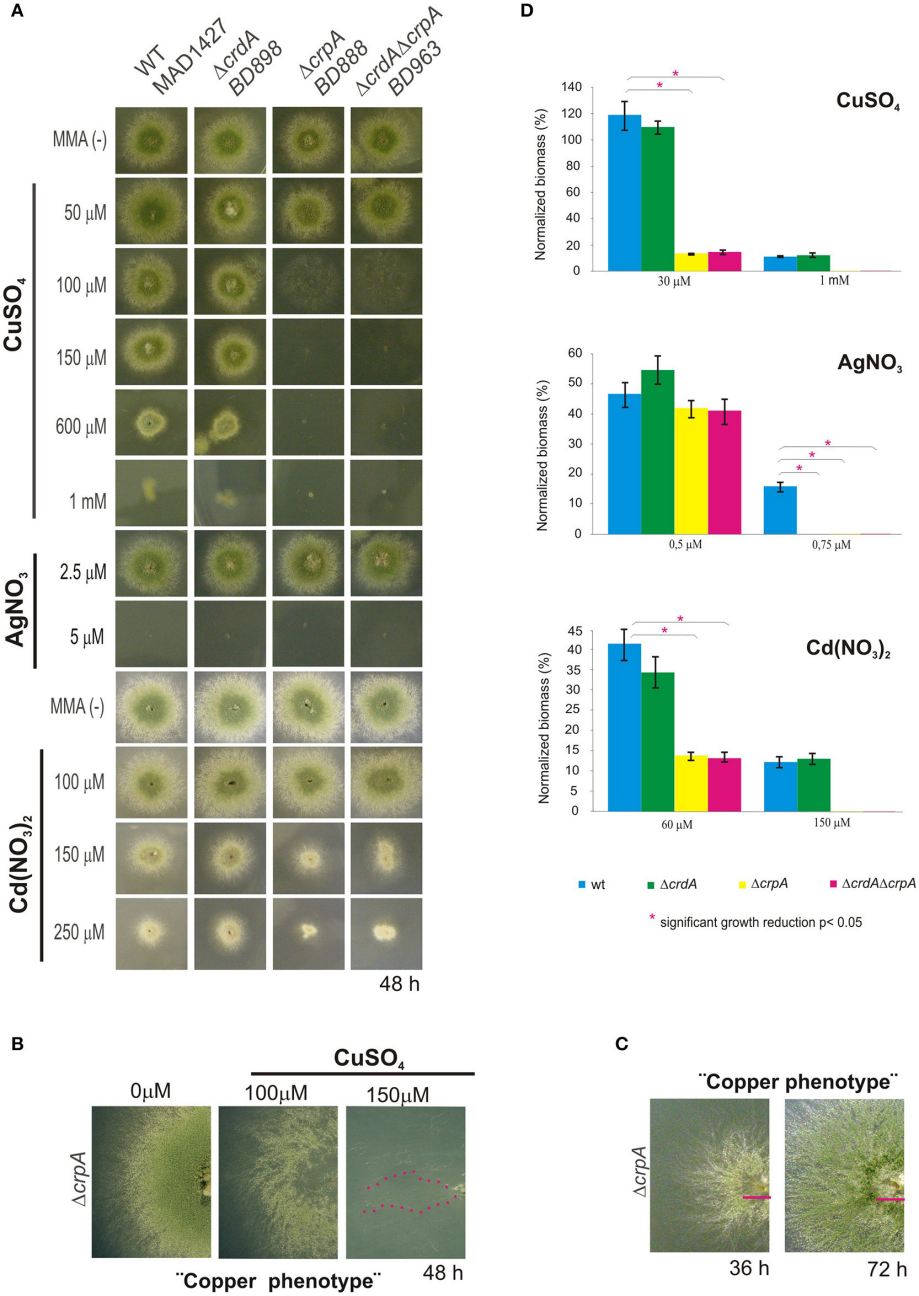
**Phenotypic analysis of *crpA* and *crdA* mutant strains**. Spores of strains having the indicated relevant genotypes were point-inoculated on standard MMA. Images of colonies were taken after 2 days of incubation at 37°C. **(A)** Mutant characterization in solid medium supplemented with indicated concentrations of metal salts **(B)** Close-up views of the morphological colony alterations in the central region of the colony caused by the deletion of *crpA* when exposed to 100 μM CuSO_4_, denominated “Copper phenotype.” At 150 μM CuSO_4_ isolated hyphae were observed (magenta dotted line). **(C)** Recovery of cellular growth in the central region of the colony over the time (magenta lines). Images of colonies were taken after 36 and 72 h of incubation. **(D)** Cellular growth in liquid MMA was monitored by determining the dry weight (biomass) of cells grown 24 h at 37°C with the indicated concentrations of metal salts. In order to facilitate data comparison growth of each strain at basal level (no stress) was designated 100%, data was normalized and presented as percentages. Graphs show the means ± standard deviation (*SD*) of triplicate experiments (*n* = 3). ^*^Significant growth reduction *p* < 0.05.

To assess the role of *crpA* and *crdA* in copper tolerance, we generated single-knockout mutants for each locus by gene replacement technique (see Section Material and Methods). The mutant Δ*crpA* (BD888), Δ*crdA* (BD898), and wild-type (WT) strains were tested at different copper concentrations. As shown in Figure 2A, the *crpA* null strain showed reduced resistance to Cu^+2^, exhibiting morphological defects at 100 μM CuSO_4_ and a nearly total growth inhibition at 150 μM CuSO_4_. At 100 μM CuSO_4_ the *crpA* deletant exhibited similar colony radial growth compared to the wild-type strain, albeit with a significantly lower cellular density in the central region of the colony (Figure 2B). We named this cellular morphology “copper phenotype,” to distinguish it from those obtained with other metal ions (see below). After additional 24 h of incubation the scarce cellular growth visualized gradually recovered resembling wild-type morphology (Figure 2C, magenta line). Close up views of strain BD888 inoculated in medium supplemented with 150 μM CuSO_4_revealed the presence of isolated hyphae with normal extension pattern across the whole colony (Figure 2B, magenta dotted line). No cellular recovery was observed after additional incubation, as described previously. The “copper phenotype” was rescued to wild-type by introduction of *A. nidulans crpA* gene in the Δ*crpA* strain, confirming the involvement of the P_I_-ATPase pump in copper tolerance (Supplementary Figure 1). In contrast, the null *crdA* mutant was not abnormally susceptible to high Cu^+2^ concentrations, displaying wild-type phenotype over the concentration range tested (Figure 2A).

To ascertain the participation of the putative metallothionein in the copper detoxification pathway we generated a double null deletant strain (BD963) lacking CrdA and CrpA. The double null Δ*crdA*Δ*crpA* and the single Δ*crpA* mutants, showed indistinguishable copper sensitivity (Figure 2A), denoting that *crdA* and *crpA* mutations were not additive. This result indicates that a functional form of CrdA is not required for resistance to toxic copper loads.

Bearing in mind the possibility that in *A. nidulans*, as it has been described in other organisms (Odermatt et al., 1994; Mandal et al., 2002), CrpA could catalyze diverse heavy-metal ion efflux, mutants were tested in plates with AgNO_3_ and Cd(NO_3_)_2_. The extreme sensitivity of *A. nidulans* to silver in solid medium, range from 2.5 to 5 μM AgNO_3_, did not allow us to determine accurate silver tolerance data. In the case of cadmium, the *crpA* null strain exhibited colony defects such as reduced (~50%) and compact growth at 150 μM of Cd(NO_3_)_2_. This colony morphology was distinct from the “copper phenotype” formerly described (Figure 2A). As previously, the *crdA* deletant did not manifest a phenotype related to heavy metal ion tolerance.

Sensitivity of mutant strains was also evaluated in liquid culture by measuring mycelial biomass. In agreement with the results obtained in solid medium cultures, it was observed that deletion of *crpA* strongly affected cellular growth in liquid medium in response to copper and cadmium, whereas a null mutation of *crdA* had no significant effect. As shown in Figure 2D, in the presence of 30 μM CuSO_4_ and 60 μM Cd(NO_3_)_2_ Δ*crpA* and Δ*crdA*Δ*crpA* mutant strains decreased biomass production significantly compared to WT, up to 15 and 14%, respectively. In line with previous observations, no significant differences were detected between the two strains carrying the *crpA* deletion. In the case of silver, all mutants were unable to grow at 0.75 μM AgNO_3_.

The results obtained with the comparison of the mutants highlight that the P_I_-type pump CrpA, as a key factor in determining copper resistance in *A. nidulans* and its function may be extended to other metals. In contrast, the contribution of CrdA in tolerance to metal ion toxicity remains to be clarified.

### Dynamic expression of CrpA and CrdA in response to metal toxicity

Earlier studies had reported that after metal loading metallothionein production is increased (Liu and Thiele, 1997; Peña et al., 1998; García et al., 2002; Ehrensberger and Bird, 2011) and the detoxifying P_I_-type ATPase activity is enhanced, either inducing expression (Riggle and Kumamoto, 2000; Weissman et al., 2000) or by modifying subcellular localization (Suzuki and Gitlin, 1999; Cobbold et al., 2002).

**Figure 3:**
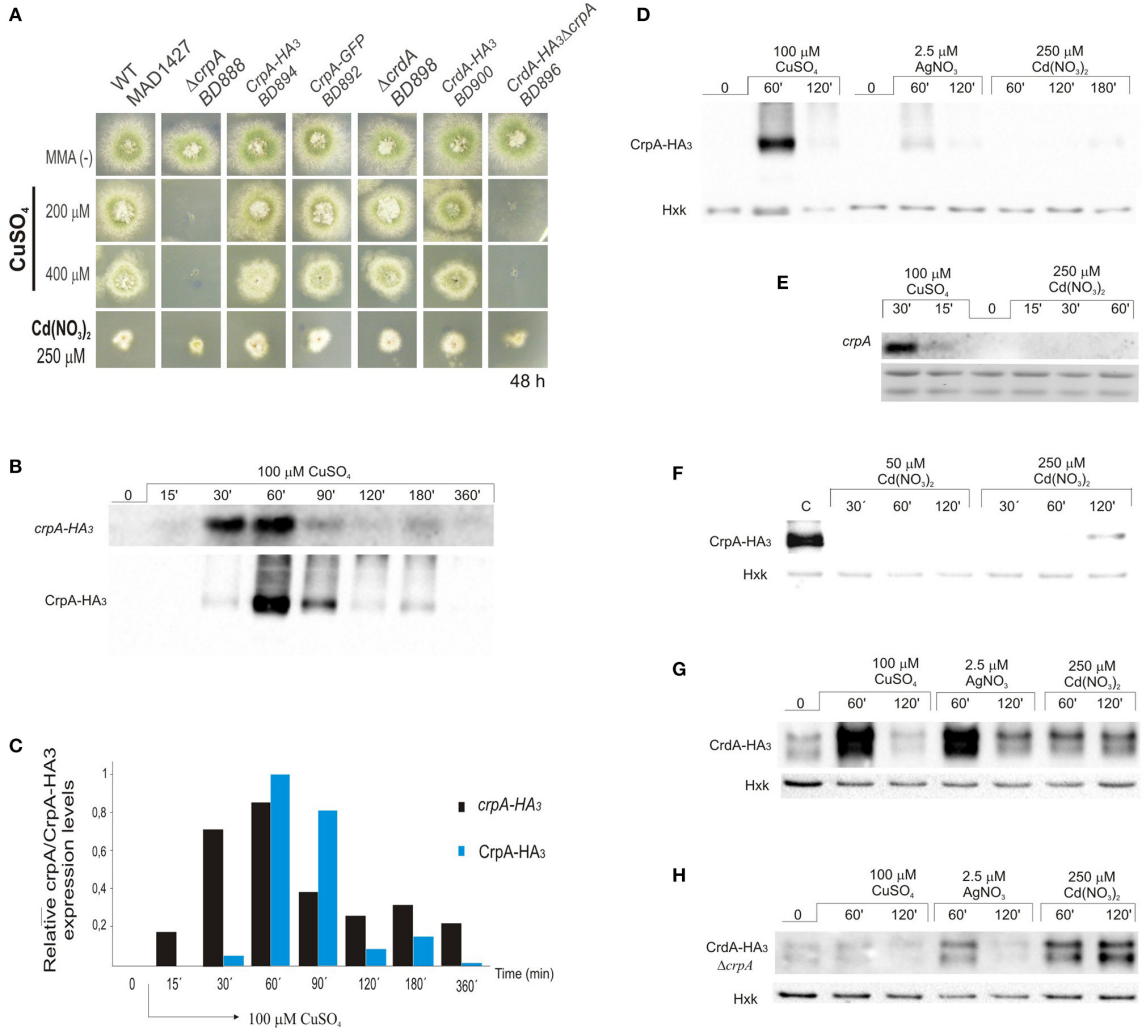
**CrpA-HA_3_ and CrdA-HA_3_ induction by heavy metal ions. (A)** Copper sensibility assay of tagged CrpA and CrdA mutant strains. **(B)** Northern blot and Western blot showing changes in *crpA-HA*_3_ transcript and CrpA-HA_3_ expression levels after addition of 100 μM of CuSO_4_ and monitored at the indicated times. Images correspond to the area of interest. **(C)** The graph shows *crpA-HA*_3_ transcript and CrpA-HA_3_ protein expressions relative to the corresponding loading controls, rRNA and hexokinase respectively. Average pixel intensity for each band was calculated with Image J (version 4.0; Fujifilm, Valhalla, NY). **(D)** Comparison of CrpA-HA_3_ induction pattern by different metal salts [100 μM CuSO_4_, 2.5 μM AgNO_3_, and 250 μM Cd(NO_3_)_2_]. Images correspond to the area of interest. **(E)** Northern blot showing changes in *crpA* expression after addition of 100 μM of CuSO_4_ but not during 1 h treatment with 250 μM of Cd(NO_3_)_2_. **(F)** Western blot comparing Cd-induced CrpA-HA_3_ expression after 50 μM and 250 μM of Cd(NO_3_)_2_ addition. Western blot showing changes in HA_3_ tagged version of CrdA in a wild-type **(G)** and null *crpA* **(H)** background induced by different metal ions.

Here we confirmed that *crpA* expression is copper-inducible. Hence, to gain a better insight into CrpA regulation during prolonged copper treatment, extracts for gene and protein expression were prepared from same mycelium samples. We generated a strain expressing a CrpA-HA_3_ fusion (BD894) and since HA_3_ tagging might alter CrpA function we tested copper and cadmium tolerance on plates. BD894 displayed wild-type phenotype indicating that the CrpA-HA_3_ chimera was functional at the copper concentrations used for gene and protein expression (Figure 3A). After spores were incubated for 16 h, cells were harvested at different times, before (0 min) and after addition of 100 μM of CuSO_4_ (15–360 min). *CrpA-HA*_3_ and CrpA-HA_3_ signals were measured and normalized with respect to rRNA and hexokinase signals.

Northern blot analysis revealed that the transcript of *crpA* was not detectable in total RNA extracts from resting cells (Figure 3B). After 15 min of Cu-addition to the culture *crpA* was quickly induced (6-fold), reaching maximum mRNA levels (57-fold) within 60 min. This was followed by a gradual repression to expression levels compared with those observed at 15 min (8-fold; Figure 3C). The mRNA levels were maintained during the 360 min that last the experiment. These results confirmed that *crpA* expression is transiently induced to high levels in response to copper toxicity, but in prolonged exposures, low basal levels are maintained. Northern blot expression patterns matched with CrpA-HA_3_ fusion protein (~134 kDa) kinetics, despite the fact that the band corresponding to the chimera was first detected 30 min after copper addition (Figure 3B). Western blot analyses additionally revealed dispersed high-molecular weight CrpA-HA_3_ species and a barely detectable smaller band within 1 h, which may reflect post-translational modification events.

To further elucidate the role of CrpA in non-copper metal ion detoxification, protein induction was studied under silver and cadmium stress conditions. It was determined that, in the presence of 2.5 μM AgNO_3_, CrpA-HA_3_ followed the expression pattern observed with copper, reaching a maximal level within 60 min followed by a decline (Figure 3D). However, the amplitude of the response was considerably lower. In contrast, 250 μM Cd(NO_3_)_2_ did not activate protein expression until 2 h after the treatment, being the band detected very faint. This finding was not consistent with the observation that the deletion of *crpA* rendered the cells more sensitive to cadmium. Therefore, we monitored protein expression in cells treated in longer period. It was then observed that protein levels were increased in the 120–180 min time range (Figure 3D).

It has been reported that high concentrations of heavy-metal ions can inhibit synthesis of certain proteins (Matts et al., 1991; Staneviciene et al., 2008). To test if this was the cause of the late CrpA detection, *crpA* expression in earlier time points was studied. The analyses showed that no Cd-induced *crpA* signal was detected after 1 h of exposure to the cation, while Cu-induced mRNA was visualized as early as 15 min after exposure (Figure 3E). To further explore this aspect, protein expression with lower cadmium concentration was studied and compared with that of high concentration. No CrpA-HA_3_ was detected over the incubation period with 50 μM Cd(NO_3_)_2_. In contrast, the protein was detected after 2 h of treatment with 250 μM Cd(NO_3_)_2_ (Figure 3F)_._

Riggle and Kumamoto (2000) pointed out that CaCrd2 was implicated in the initial buffering of copper ions. Since the possible function of CrdA was studied after 2 days of culture in solid medium we decided to assess its role at earlier times by protein expression analysis. The putative metallothionein expression was observed even in the absence of metals. A band of ~15 kDa, similar to the predicted size of the HA_3_ tagged version of CrdA, and a second slower-migrating band of ~17 kDa were detected. The relative levels of both bands varied under different conditions and experiments. As it is observed in Figure 3G protein levels were increased in response to all metal ions tested. Once more, CuSO_4_ and AgNO_3_ triggered a similar protein expression pattern, although the effect of silver on CrdA-HA_3_ was more robust than on CrpA-HA_3_, being comparable to copper induction. Unlike the previous case, cadmium elicited protein expression by the first hour of treatment and remained at same level throughout the time course of the experiment. This response was faster and stronger than the Cd-induction observed in copper ATPase.

We then hypothesized that the absence of CrpA, which plays the principal role in copper resistance and was highly expressed at this time point, may lead to an enhance of CrdA expression in order to augment Cu-buffering. To test this possibility, a Δ*crpA* mutant strain carrying *crdA-HA*_3_ construct was generated (BD896; Figure 3A). Comparing the signal of the bands corresponding to CrdA-HA_3_ in the wild-type and null *crpA* background, in the last case, intensities detected were much lower for Cu^+2^ and Ag^+^, and approximately similar for Cd^+2^ (Figure 3H). This result indicates that the absence of *crpA* negatively affect CrdA expression.

In summary, these data show that *crpA* expression fluctuates in a prolonged copper treatment, being strongly and transiently induced as an early response, but lightly and continuously maintained thereafter. In addition, the results strongly suggest that copper is the principal ion transported by CrpA but has a role in silver transport. In addition, CrdA is involved in the early response to metal toxicity, but its role remains to be further elucidated.

### Localization of CrpA-GFP at the cellular surface

**Figure 4:**
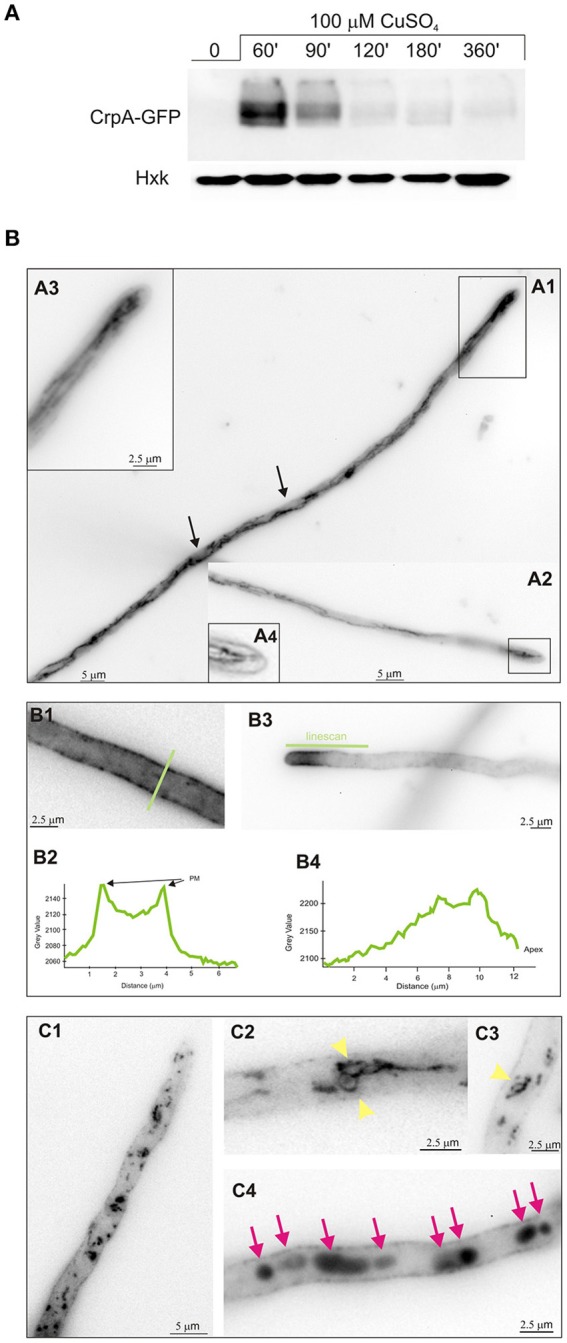
**Effect of copper in intracellular localization of CrpA-GFP**. **(A)** Western blot confirming CrpA-GFP normal expression kinetics after addition of 100 μM of CuSO_4_ **(B)** Cells of a strain expressing a CrpA–GFP fusion were grown in selective medium for microscopy for 16 h at 25°C and shifted to medium containing 100 μM CuSO_4_ for the indicated times. Images taken 30 min after the shift. **(A1,A2)** CrpA localized in a network of strands and tubules. **(A3,A4)** Images corresponds to the rectangular region indicated in **(A1,A3)** showing a magnification of the tip region. **(B1–B4)** Images corresponding to 1 h after the shift. GFP fluorescence was accumulated predominately in the PM **(B1)** and polarized in the tip region **(B3)**. Panels **(B2,B4)** corresponds to the line scans of CrpA-GFP signal across the indicated lines. **(C1–C4)** Images taken 2–3 h after shifting. Panels **(C1,C2,C3)** are examples of ring-shaped structures (yellow arrowheads) and **(C4)** of abnormal aggregates (magenta arrows). Images were treated with sharp filter, shown in inverted gray contrast and represent average intensity projections of z-stacks.

Considering that the biological activity of copper P_I_-ATPase is likely to extrude copper from the cell, we hypothesized that CrpA localizes in the plasma membrane. Cellular distribution of CrpA protein was investigated with a C-terminus GFP-tagged protein. To determine whether the CrpA-GFP fusion protein was functional, the strain expressing it (BD892) was grown in standard minimal medium with copper and cadmium stress and, as expected, displayed wild-type phenotype (Figure 3A). Functionality was also assessed studying protein expression by Western blotting using mouse anti-GFP antibody (Figure 4A), showing HA_3_-fusion protein kinetics.

For life image experiments, cells were grown in minimal liquid medium for microscopy without copper stress and shifted to media containing 100 μM CuSO_4_. As observed by epifluorescence microscopy, upon 30 min of the shift CrpA localized to a reticulated network of interconnecting tubules, surrounding structures that likely represent nucleus (Figures 4B.A1,A2, arrowed), and strands associated to the plasma membrane that reminded to the ER (Markina-Iñarrairaegui et al., 2013). Close up views of the tip region (boxed area) showed (Figures 4B.A2–B.A4) a finger-like protrusion directed toward the tip. Thirty minutes later CrpA distribution was modified, showing a homogeneously dispersed localization in the cytoplasm and a strong fluorescence across the entire length of the cell periphery, suggesting CrpA-GFP protein predominated at the plasma membrane (PM; Figures 4B.B1,B2), consistent with its predicted function. In a large number of cells CrpA was polarized in the proximity of the tip of hyphae (Figures 4B.B3, B4). Fluorescence was measured and represented in graphs corresponding to the linescans across the indicated lines, verifying signal accumulation in the proximity of the plasma membrane and tip region.

Figures 4B.C1 illustrates that 2 h after copper load, even if a significant proportion of CrpA-GFP appears to remain at the PM, it was also distributed in dispersed structures of variable sizes and morphology, punctuated and ring-shaped structures, throughout the cytosol (Figures 4B.C2,C3, yellow arrowheads) akin to Golgi equivalents (Pantazopoulou and Peñalva, 2009). An hour later large aggregates were noticeable along the hyphae that look like vacuolar compartments (Pantazopoulou et al., 2007; Figures 4B.C4, magenta arrows). Nevertheless, the identity of these membrane organelles has to be elucidated and further studied by colocalization experiments.

Overall, these results suggest that *de novo* CrpA translocates to the cell surface, most certainly to efflux copper, leaves the plasma membrane and reaches different organelles as part of an orchestrated trafficking process.

### Expression regulation of CrpA and CrdA by the transcription factor AceA

The rapid and regulated copper-inducible transcription of *crpA* suggested that it is under the control of a copper-dependent transcription factor (TF). Blastp searches using *S. cerevisiae* ScAce1p (CAA96877.1; Dameron et al., 1991), *Candida glabrata* CgAmt1p (XP_447430.1; Zhou et al., 1992), and *Yarrowia lipolytica* YlCrf1p (XP_500631.1; García et al., 2002) amino acid sequences allowed the identification of the putative transcription factor homologous in *A. nidulans*, a 525 amino acid long protein encoded by AN1924 ORF (1,689 bp) and referred to from now on as AceA.

**Figure 5:**
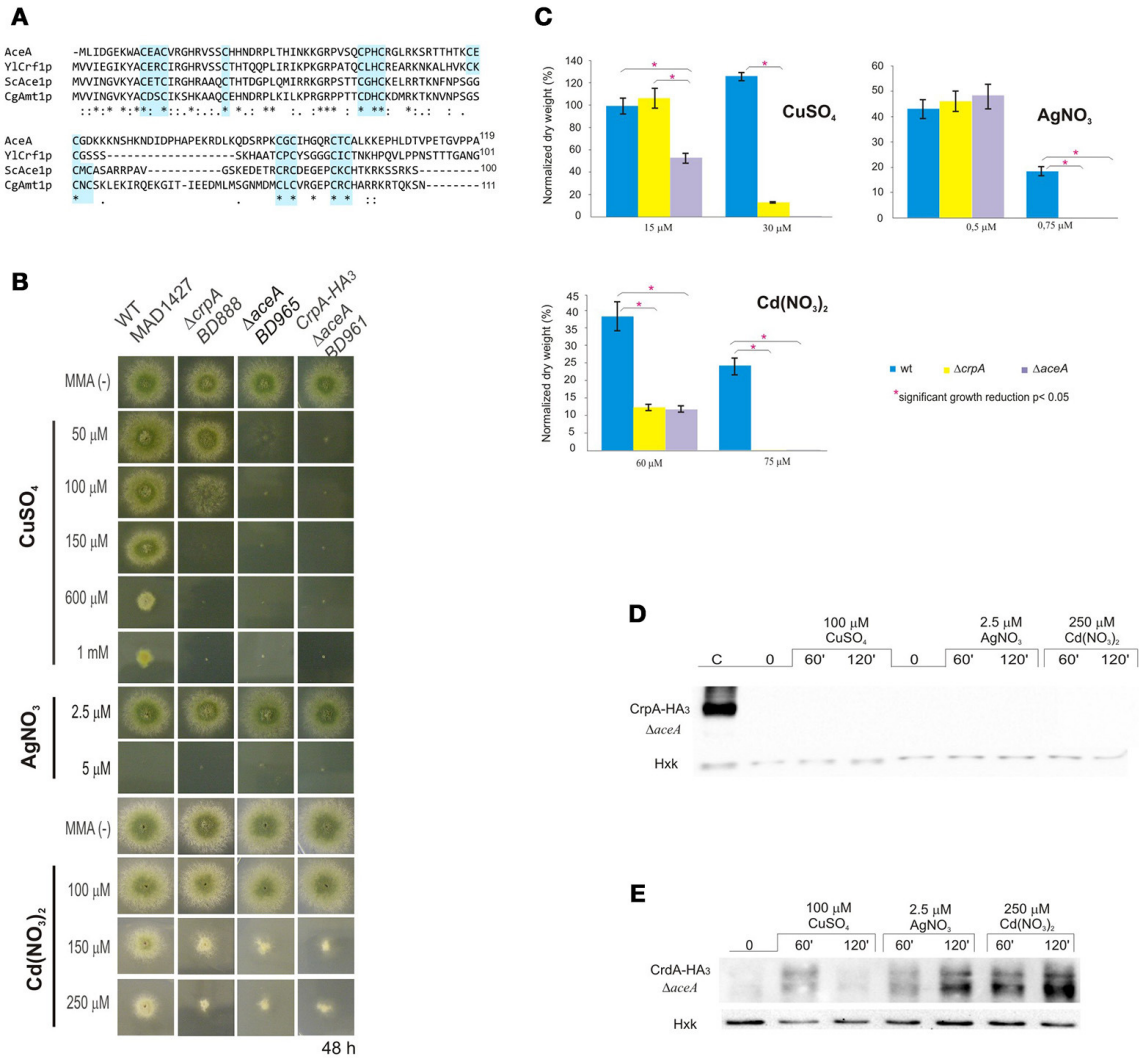
**Functional analysis of AceA. (A)** Multiple alignment of the most conserved region, N-terminal half, of AceA, YlCrf1p, ScAce1p, and CgAmt1p, which contains the majority of cysteine residues arranged in clusters (indicated in light blue boxes). Protein alignments were performed using Clustal Omega. Genebank protein accesion numbers are as follows: *A*. *nidulans* AceA (CBF85835.1), *Y. lipolytica* YlCrf1p (XP_500631.1), *S. cerevisiae* ScAce1p (CAA96877.1), and *C. glabrata* CgAmt1p (XP_447430.1). **(B)** Δ*aceA* mutant characterization in solid medium supplemented with indicated concentrations of CuSO_4_, AgNO_3_, and Cd(NO_3_)_2_. **(C)** Biomass measurement of WT, Δ*aceA* and Δ*crpA* strains grown at indicated conditions. Data was normalized and presented as percentages. Bars indicate means and error bars standard deviation. *N* = 3. Western blot showing **(D)** CrpA-HA_3_ and **(E)** CrdA-HA_3_ induction by heavy metal ions in a null *aceA* background strain. Hexokinase and CrpA-HA_3_ sample of cells treated for 1 h with copper were used as controls. ^*^Significant growth reduction *p* < 0.05.

AceA contains several features described for its orthologs, especially the N-terminal DNA-binding domain. This region (Figure 5A) showed the highest sequence conservation, especially the first subdomain (residues 1–39), copper-fist DNA-binding domain, which displayed 61% identity with 1–40 amino terminal amino acids of YlCrf1p (76% similarity), 55% with ScAce1p (73% similarity) and 45% identity with CgAmt1p (76% similarity). This whole region encompasses 11 of the 14 encoded cysteines arranged in CxC or CxxC clusters, conserved among the copper regulatory transcription factors mentioned. These cysteine residues are necessary for copper binding which induces a conformational change of the protein. This, in turn, allows the interaction of the copper-activated DNA-binding region with the Metal Regulatory Elements (MRE) of the promoter of the target genes (Peña et al., 1998). AceA exhibits a serine (14%) and proline (12%) rich composition and unusual distribution (SSxSS, SSS, PPP clusters), especially in the C-terminal half of the protein, similar to YlCrf1p of *Y. lipolytica* (García et al., 2002).

The conservation of motifs suggests that AceA may be the transcriptional factor involved in copper tolerance in *A. nidulans*. To investigate its role, a single-knockout mutant of *aceA* locus was generated (BD965) and tested in the same range of metal concentrations used before. Growth analyses demonstrated that not only did the Δ*aceA* strain display an identical “copper phenotype” to that observed in Δ*crpA*, but it also exhibited a more pronounced sensitivity response to CuSO_4_, since colony defects were observed at lower concentration (Figure 5B). Elevated cadmium content provoked a reduction of colony growth, nevertheless in a similar degree to the previously observed in the Δ*crpA* strain. Biomass measurements supported these results (Figure 5C). The Δ*aceA* mutant strain was significantly more sensitive to CuSO_4_ than Δ*crpA* strain (*P* < 0.05). In contrast, the tolerance to Cd^+2^ and Ag^+^ was almost identical in both strains. These results confirm the implication of AceA in metal tolerance, playing a key role in copper resistance.

We hypothesized that AceA was the copper dependent transcriptional activator of genes involved in copper detoxification, thus the strain bearing a mutation in the *aceA* gene should be sensitive to copper primarily due to a defect in copper-inducible transcription. In order to prove this hypothesis, we analyzed by Western blot extracts of strain BD961, which expressed the CrpA-HA_3_ fusion protein in an *aceA-*deletant background (Figure 5B). Using anti-HA_3_ antibody, we did not detect the 134 kDa band observed previously, as in extracts from the strain expressing CrpA-HA_3_ and loaded as control (Figure 5D). The lack of CrpA-HA_3_ detection demonstrated that AceA is necessary for metal induction of the P_I_-type pump transcription. Subsequently, the HA_3_ tagged metallothionein-like protein in response to metal salts in the absence of *aceA* allele (BD1062) was analyzed. CrdA-HA_3_was detected in the Δ*aceA-*deleted strain (Figure 5E). Moreover, results indicated that CrdAexpression levels and kinetics in the null Δ*crpA* and Δ*aceA* mutant strains were comparable. This result indicates that AceA is not essential for metal-induced activation of *crdA* and supports the possibility of a role different from metal ion detoxification.

Taken together, these results confirm that AceA is the transcription factor responsible of regulating genes involved in copper detoxification, as CrpA, and suggest the existence of an as yet unidentified gene responsible for the residual copper resistance in *A. nidulans*.

## Discussion

Eukaryotes maintain physiological copper levels by regulating the balance between copper uptake, compartmentalization and detoxification. In this study, we identified and characterized two key operators of the copper detoxification system of *A. nidulans*, which relies principally in excretion.

### CrpA is a putative copper P_I_-type ATPase

Sequence analysis identified CrpA as the putative homolog of *C. albicans* CaCrp1p, which had been reported to confer high resistance to copper by ion extrusion (Riggle and Kumamoto, 2000; Weissman et al., 2000). CrpA and CaCrp1p share an atypical structure and distribution of the N-terminal metal binding domains (MDB). Since these motifs are likely involved in regulation, we postulate that CrpA and CaCrp1p have similar ion specificities and biological function. Studies carried out in *Y. lipolytica* strongly support the idea that copper resistance is not mediated by MT related Cu buffering but by efflux (García et al., 2002; Ito et al., 2007). Additional blast searches in this dimorphic fungus revealed a putative homolog, YALI0B02684p (GeneBank accession number XP_500433.1), which displayed a similar NH_2_-terminal extension. These results indicate that this feature is not unique to *A. nidulans* and *C. albicans* and it could be characteristic to fungal species that utilize the described ATPase-based extrusion system as the primary way to overcome high copper loads. It is also important to underscore the crucial role of copper resistance for virulence of clinically important fungal pathogens as *C. albicans* (Mackie et al., 2016), *Cryptococcus neoformans* (Samanovic et al., 2012; Ding et al., 2013) and *A. fumigatus* (Dietl et al., 2016; Wiemann et al., 2017).

### Copper detoxification relies on the P_I_-type ATpase CrpA

Evidence of the direct participation of CrpA in copper resistance was obtained by the extreme sensitivity exhibited by CrpA null mutants. An atypical aspect of the phenotype of the null *crpA* mutation was the poor growth in the central region of the colony that overcame toxicity with time. This adaptive behavior could be partially explained by the growth pattern described by Valix et al. (2001). As the colony matures, copper may bind to new cellular material locally, resulting in reduction of free copper concentration, and increased growth.

Besides copper, Δ*crpA* also exhibited sensitivity to cadmium, however the divergent phenotype reflected either affinity of metals for different targets and/or distinct detoxification paths (Mendoza-Cózatl et al., 2010). Neither *crpA* Cd-induction nor Cd-protein expression under low Cd concentration were observed. In addition, CrpA was detected only in the presence of high cadmium loads. Together, these results support an indirect P_I_-type ATPase activation taking place, possibly due to the saturation of the main cadmium detoxification system, a Cd^+2^ pump (Shiraishi et al., 2000; Thorsen et al., 2009) or glutathione-derived peptides denominated phytochelatins (Mendoza-Cózatl et al., 2005, 2010).

Regarding silver toxicity, our data showed that *A. nidulans* is sensitive to this metal at a very low (2.5–5 μM) concentration range. Biomass measurements indicate that CrpA may participate in silver resistance. On the other hand, it was observed that silver induced expression of CrpA which matched copper kinetics, although to a lesser extent. Based on these results, we propose that copper is the principal substrate of the *A. nidulans* ATPase, however silver can also be transported. Indeed, this is consistent with the fact that orthologs described above are also reported Cu^+^/Ag^+^ transporters. Although we did not ascertain it in this study, CrpA likely exports Cu^+^ and not Cu^+2^ since; (a) in the reduced cytosolic environment Cu^+2^ is found in Cu^+^ form and (b) substitution of Cu^+^ by Ag^+^ is possible in proteins with CxxC containing MBDs, but not Cu^+2^ (Petris et al., 1996).

Long term copper exposure induces an early, strong, and transient expression followed by a basal maintenance expression level of CrpA. Considering the effect of copper exposure on *crpA* expression over time, we demonstrated that *crpA* induction is rapid (within 15 min), transiently strong (57-fold increase in 1 h) and sustained throughout the exposure to copper, since mRNA levels were detectable along the whole experiment. Hypothesizing that *crpA* mRNA levels may reflect intracellular ion concentrations, we consider that under long term high copper exposure, cells would firstly synthesize an elevated amount of Cu-exporter in order to rapidly reduce the initial burst of copper incorporated via high affinity transporters. Subsequently, pump levels would be downregulated and then maintained at a certain level during the period in which copper continues to enter chronically to the cell via low affinity transporters, similar to *S. cerevisiae* (Yu et al., 1996). Regulation studies are ongoing at present in our laboratory to verify whether CrpA post-translational modifications (PTM) are involved in CrpA activity control besides the transcriptional regulation described in this work.

### CrpA localizes in the plasma membrane

CrpA localization close to the cell surface in response to copper addition sustains the expected copper extrusion role of this ATPase. Detailed evaluation of the subcellular distribution indicated that CrpA is under the control of a highly orchestrated trafficking process that resembles membrane transporter regulation (Pantazopoulou and Diallinas, 2007; Lauwers et al., 2010). We speculate, that in response to increased cytosolic Cu^+^, *de novo* synthesized CrpA traffics from the ER to the Golgi compartment and then to the plasma membrane (PM). Upon a purported decrease in cytosolic copper concentration, a fraction of PM CrpA would be endocytically internalized and sorted to the multivesicular body (MVB). CrpA could then be recycled back to the PM via the TGN as reported in human cells (Pase et al., 2004), where PM CrpA could be the result of two different protein pools; a newly synthesized and a recycled CrpA pool. Post-traslational modification and organelle trafficking mutant studies would be required to establish a model of CrpA sorting and regulation. Solving the molecular mechanism of the P_I_-type ATPase trafficking in *A. nidulans* could provide novel insights into how cells with a polarized architecture regulate copper ATPase activity.

### The transcription factor AceA regulates metal-responsive CrpA expression

The active role of AceA in copper resistance gene regulation was demonstrated by the extreme copper-sensitive phenotype of the knock-out mutant and the absence of expression of CrpA in the Δ*aceA* strain. In fact, the increased sensitivity of Δ*aceA* with respect to Δ*crpA* mutants highlighted two major points: firstly, the participation of an additional element in the copper detoxification path and secondly, the predominant role of CrpA over the minor participation of the other player in the system. This second participant could be either a copper-metallothionein (MT) that has not been identified or a copper scavenging phytochelatin, like in *Schizosaccahoromyces pombe* (Clemens et al., 1999). Identification of new metallothioneins, since the primary structure is highly diverse, could be a laborious task. It may be worthy to attempt a different approach. The characterization of the AceA binding metal-response element (MRE) on the CrpA promoter might propose a consensus MRE sequence for *A. nidulans* copper detoxification genes that could facilitate new searches.

### CrdA, a putative metallothionein with undefined function

CrdA expression was induced by copper, silver and cadmium within 1 h, indicating a role in metal homeostasis. In summary, we found that: (a) a Δ*crdA* strain did not exhibit a metal sensitivity phenotype, (b) a non-additive effect of *crpA* and *crdA* deletion was observed, (c) the deletion of *crpA* did not increase *crdA* transcription, and (d) AceA was not required to activate CrdA expression. Taken together, these results do not support the participation of CrdA in CrpA-mediated metal detoxification. A likely role for this putative metallothionein may be as an antioxidant (Palmiter, 1998; Blindauer and Leszczyszyn, 2010). Further studies will confirm whether the principal AceA-regulated P_I_-type ATPase (CrpA) system is connected with CrdA mechanisms in response to metal stress.

## Author contributions

MA conducted the experimental work, analyzed data, and contributed to the writing of the paper. AM made the concept and designed the work, analyzed, and interpreted the results, wrote the manuscript, and ensured the accuracy of the project as whole. UU co-conceived the work, ensured the scientific issue was appropriately investigated, ensured the integrity of the work, revised, and approved the final version to be published.

## Funding

This work has been supported by the Basque Government through the grants IT599-13 and S-PC13UN041 to UU. Publication fees of the work has been co-financed by Frontiers Publishing Grants.

### Conflict of interest statement

The authors declare that the research was conducted in the absence of any commercial or financial relationships that could be construed as a potential conflict of interest.

## Supplementary material

Supplementary Figure 1 ***crpA* rescue phenotype**. WT, Δ*crpA*, and *crpA*^+^ complemented mutant strain characterization in solid medium supplemented with the indicated CuSO_4_ concentrations.
