# Supplementary figures and images for: Copper Resistance in Aspergillus nidulans Relies on the PI-Type ATPase CrpA, Regulated by the Transcription Factor AceA
Source: Front Microbiol. 2017 May 30;8:912. doi: 10.3389/fmicb.2017.00912 (PMC5447758; doi:10.3389/fmicb.2017.00912)

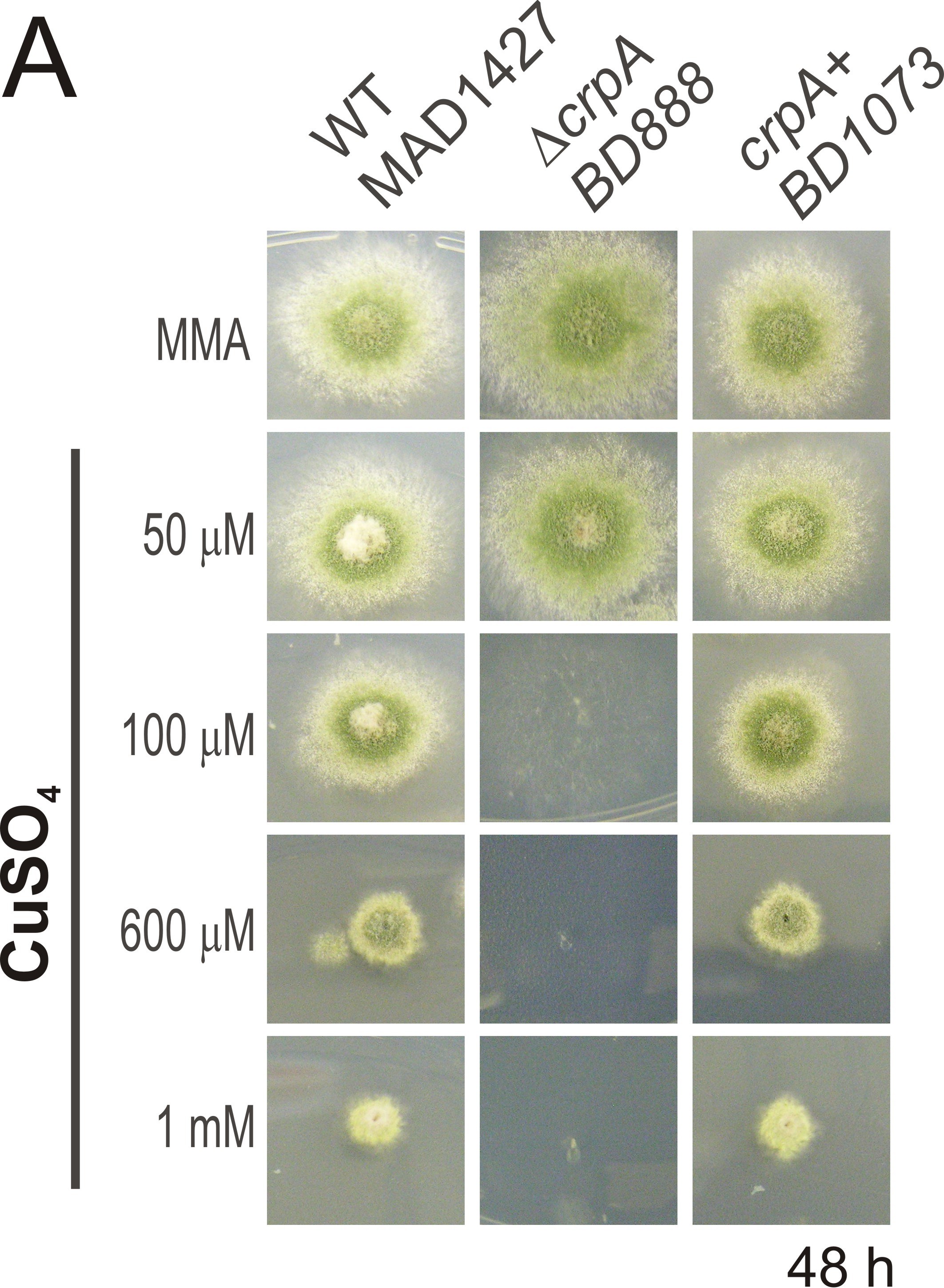

Supplement: Supplementary Figure 1 — crpA rescue phenotype. WT, ΔcrpA, and crpA+ complemented mutant strain characterization in solid medium supplemented with the indicated CuSO4 concentrations. [file Image1.JPEG]
